# Salvage treatment for refractory or relapsed acute myeloid leukemia: a 10-year single-center experience

**DOI:** 10.6061/clinics/2020/e1566

**Published:** 2020-04-06

**Authors:** Wellington Fernandes da Silva, Lidiane Inês da Rosa, Fernanda Salles Seguro, Douglas Rafaele Almeida Silveira, Israel Bendit, Valeria Buccheri, Elvira Deolinda Rodrigues Pereira Velloso, Vanderson Rocha, Eduardo M Rego

**Affiliations:** I Instituto do Cancer do Estado de Sao Paulo (ICESP), Hospital das Clinicas HCFMUSP, Faculdade de Medicina, Universidade de Sao Paulo, Sao Paulo, SP, BR; II Hematologia, Hospital das Clinicas HCFMUSP, Faculdade de Medicina, Universidade de Sao Paulo, Sao Paulo, SP, BR

**Keywords:** Acute Myeloid Leukemia, Salvage Regimens, Prognostic Factors, Survival, Cohort Study

## Abstract

**OBJECTIVES:**

The outcomes of refractory and relapsed acute myeloid leukemia (AML) patients in developing countries are underreported, even though the similar classic regimens are widely used.

**METHODS:**

We conducted a retrospective comparison of “MEC” (mitoxantrone, etoposide, and cytarabine) and “FLAG-IDA” (fludarabine, cytarabine, idarubicin, and filgrastim) in adults with first relapse or refractory AML.

**RESULTS:**

In total, 60 patients were included, of which 28 patients received MEC and 32 received FLAG-IDA. A complete response (CR) rate of 48.3% was observed. Of the included patients, 16 (27%) died before undergoing bone marrow assessment. No statiscally significant difference in CR rate was found between the two protocols (*p*=0.447). The median survival in the total cohort was 4 months, with a 3-year overall survival (OS) rate of 9.7%. In a multivariable model including age, *fms-like tyrosine kinase 3* (*FLT3*) status, and stem-cell transplantation (SCT), only the last two indicators remained significant: *FLT3-ITD* mutation (hazard ratio [HR]=4.6, *p*<0.001) and SCT (HR=0.43, *p*=0.01).

**CONCLUSION:**

In our analysis, there were no significant differences between the chosen regimens. High rates of early toxicity were found, emphasizing the role of supportive care and judicious selection of patients who are eligible for intensive salvage therapy in this setting. The *FLT3-ITD* mutation and SCT remained significant factors for survival in our study, in line with the results of previous studies.

## INTRODUCTION

Acute myeloid leukemia (AML) is a highly heterogeneous disease in adults that is fatal in the majority of patients 1. It is estimated that only 15%–40% of patients achieve long-term survival with current approaches, which include genetic risk stratification and allogeneic stem-cell transplantation (SCT) for intermediate-high risk subjects 2,3. Although toxicity is a major concern when treating AML in adults, the high refractoriness and rate of relapse seems to be the leading cause of death, even in developing countries 4,5.

There is no consensus regarding the best salvage regimen for refractory or relapsed AML (r/rAML); the regimen is traditionally based on high-dose cytarabine in a changeable combination with anthracyclines, purine analogs, and etoposide 6,7. The recent incorporation of innovative drugs such as gemtuzumab, ozogamicin, hypomethylating agents, and *fms-like tyrosine kinase 3* receptor (FLT3)-inhibitors has improved outcomes in this setting, but these approaches are not widely available 8-10. Furthermore, the reported complete response (CR) rates vary from 15% to 65% following the SCT procedure, which is reported to be an essential step toward achieving long-term remission 6.

The outcomes of r/rAML patients in developing countries are underreported, even though the similar regimens are widely used. We conducted a single center retrospective comparison of two regimens, “MEC” (mitoxantrone, etoposide, and cytarabine) and “FLAG-IDA” (fludarabine, cytarabine, idarubicin, and filgrastim), in the adult population with refractory or first relapse of AML, with the aim to describe this population and their outcomes.

## MATERIALS AND METHODS

### Study design and ethics statement

This was a retrospective single-center study conducted at the Institute of Cancer of São Paulo (ICESP), University of São Paulo (USP), in Brazil. Clinical and laboratory data were obtained from the databases of the Leukemia Clinic of Discipline of Hematology. All procedures were in accordance with the ethical standards of the institutional research committee (CAPPEsq – CAAE: 80673316.3.0000.0068) and with the 1964 Helsinki Declaration and its later amendments, or comparable ethical standards.

### Patients

All patients aged above 16 years who received MEC or FLAG-IDA between December 2009 and January 2019 were initially included. Patients who received one of the above regimens as first-line therapy or patients with other diagnoses besides non-promyelocytic AML were excluded. Patients who did not receive the salvage regimen at our center were also excluded from this study.

The AML diagnosis was established based on the World Health Organization criteria, using morphology, immunophenotyping, and conventional karyotyping in all cases. Screening for *NPM1* and *FLT3* mutations was performed in all cases by standardized methods 11. *CEBPA* mutations and *BCR-ABL1* fusion were heterogeneously screened. Some cases had their genetic evaluation complemented by FISH if necessary. Clinical variables were retrospectively collected and managed using REDCap electronic data capture tools hosted at the University of São Paulo 12.

### Definitions, treatments, and response evaluation

In our center, the local protocol recommended a “7+3” classical regimen for first-line remission induction in fit patients with AML, which involves daunorubicin, idarubicin, or mitoxantrone as the anthracycline/anthracenedione 13. Response is assessed 7–14 days after the end of induction, as classically recommended 14. Patients who did not achieve partial remission (50% reduction in bone marrow [BM] blasts, resulting in less than 25% blasts), CR (absence of extramedullary leukemia, <5% blasts in the BM, absence of circulating blasts or blasts with Auer rods, and platelet count ≥100×10^9^/L and neutrophil count ≥1.0×10^9^/L), or CRi (same criteria for CR, except that incomplete recovery of blood count was allowed) were considered refractory to the first-line regimen 13,14. Patients who achieved partial remission after one cycle received a second “7+3” cycle at the discretion of the physician 13. Relapse was defined as the reappearance of blasts post-CR in the peripheral blood or BM or as extramedullary disease post-CR 14. Only patients with refractory or relapsed disease following standard upfront therapy were included in this analysis and were classified into the following groups: refractory, early relapsed (relapse within 1 year from the first CR), and late relapsed (relapse after 1 year of remission). Only the first salvage regimen was considered in this study. Presumably, all patients were referred to undergo SCT as consolidation therapy after the salvage regimen at the discretion of the physician.

Patients were grouped according to their cytogenetic risk as it follows: (1) favorable: presence of *RUNX1-RUNX1T1* or *CBFB-MYH11* gene fusions or an isolated *NPM1* mutation; and (2) unfavorable: presence of an isolated *FLT3* mutation, cytogenetic abnormalities involving chromosomes 5 or 7, lysine methyltransferase 2A (*KMT2A*) rearrangement, complex karyotype (≥3 chromosomal abnormalities excluding those with favorable-risk fusions), *BCR-ABL1* fusion on real time-polymerase chain reaction (RT-PCR) or conventional karyotype, or AML cases secondary to therapy or secondary to previously known myeloid neoplasm. The remaining cases were categorized to have intermediate genetic risk.

Historically, our institution has recommended MEC as a standard therapy for r/rAML. Over the past several years, FLAG-IDA became an option for some physicians based on personal experience and the fluctuating availability of some drugs in our country. Salvage regimens were administered as previously reported: (1) MEC - mitoxantrone 6 mg/m^2^/day IV on days 1–6, etoposide 80 mg/m^2^/day IV on days 1–6, and cytarabine 1 g/m^2^/day IV on days 1–6 15; (2) FLAG-IDA - 30 mg/m^2^/day fludarabine on days 1–5, 2 g/m^2^/day cytarabine on days 1–5, and 300 mcg/day of filgrastim on days 0–5 16. Patients with Philadelphia-positive *de novo* AML were allowed to receive a tyrosine-kinase inhibitor along with the salvage regimen if indicated.

Bacterial colonization was determined weekly in all patients by rectal or perineal swabs during the induction phase and defined as isolation of any bacteria in the absence of clinical findings of infection. Invasive fungal infection (IFI) was defined as any clinical evidence of fungal disease, such as a suggestive lung nodule or necrotizing sinusitis, or fungus isolation in the blood or bronchoalveolar lavage. Serum galactomannan was also used as an ancillary test for this diagnosis. Febrile neutropenia and other complications were managed at the discretion of the physician or managed using filgrastim during BM aplasia according to local protocols.

### Statistical Analysis

Categorical variables were summarized as frequencies and percentages, and continuous variables were summarized as median and range. Pairwise comparisons between patient subgroups were performed by the Mann-Whitney test for continuous variables and by Pearson's chi-square or Fisher's exact test for categorical variables. Event-free survival (EFS) was calculated from the time of treatment initiation until the date of no response, relapse, or death. Overall survival (OS) was calculated from the time of treatment initiation until death. Survival curves were plotted with the Kaplan-Meier method and compared using the log-rank test. The median follow-up time was estimated by reversing the codes for the censoring indicator in the Kaplan-Meier analysis. The cumulative incidence of relapse (CIR) was calculated considering death as a competitor and compared by Grey's test 17. In an attempt to equalize both groups according to baseline characteristics, logistic regression was used for propensity score calculation, including for age, indication for salvage treatment, and *FLT3* status. Propensity score analysis with 1:1 matching was performed with the nearest neighbor matching method using calipers of width equal to 0.25 of the standard deviation of the logit of the propensity score to balance the baseline differences between cohorts. Factors associated with CR were investigated by logistic regression, and factors associated with survival endpoints were investigated by Cox regression. All analyses were performed using Statistical Software for Social Sciences version 22.0 (SPSS, Chicago, IL) and R software package version v 3.5.1 (R Foundation for Statistical Computing; www.r-project.org). A two-sided *p*-value <0.05 was considered statistically significant.

## RESULTS

### Patients

In our database, we identified 70 AML patients who received MEC or FLAG-IDA from 2009 to 2019. Four patients were excluded due to misdiagnosis (three had blastic phase chronic myeloid leukemia and one had acute lymphoblastic leukemia), four received FLAG-IDA as a first-line therapy for AML instead of “7+3”, and two were excluded because they had received a hypomethylating agent as first-line therapy for AML. Therefore, 60 patients were included in the final analysis.

Most patients were female (52%) with a median age of 45 years (range, 17–69). There were no cases of therapy-related AML in this cohort. Four AML cases (7%) were secondary to myeloproliferative neoplasm (MPN) or myelodysplastic syndrome (MDS). A white blood cell (WBC) count above 30×10^9^/L was present at diagnosis in 45% of cases. Regarding the genetic characterization, the karyotype was abnormal in 50% of cases. Core-binding factor alterations, namely *RUNX1-RUNX1T1* and *CBFB-MYH11* fusions, were found in 5% and 3% of subjects, respectively. In total, 22% of patients had an *NPM1* mutation, with an associated *FLT3* internal tandem duplication (ITD) present in the majority (7/13 NPM1-mutated cases). All *FLT3-ITD* positive cases had an accompanying NPM1 mutation. Two cases of Philadelphia-positive *de novo* AML were also found. Two patients had chronic human immunodeficiency virus infection and also received antiretroviral therapy. The baseline characteristics of the whole cohort are summarized in Table 1.

### Previous treatments and salvage regimens

All patients received a standard upfront regimen (“7+3”), including daunorubicin (89%), idarubicin (8%), or mitoxantrone (3%). Three patients underwent SCT in the first CR and were post-SCT relapses. Among the included patients, 43% received the salvage regimen due to refractoriness to the first-line therapy. The remaining subjects were early or late-relapsed (45% and 12%, respectively).

In total, 28 patients received MEC and 32 received FLAG-IDA in this cohort. The two subjects with Philadelphia-positive *de novo* AML in the MEC arm received dasatinib concomitantly. By comparing the baseline characteristics of both groups, no significant differences were found in age, sex, initial WBC count, bacterial colonization, genetic risk, and *FLT3* status. When it comes to the indication for salvage treatment, there were more refractory cases in FLAG-IDA group (56% *vs*. 28%, *p*=0.029) (Table 2).

### Responses and survival data

Considering the whole cohort, 17/60 achieved CR and 12/60 achieved CRi, with a total CR rate (CR+CRi) of 48.3% (95% confidence interval [CI], 35.4–61.5). In total, 16 patients (27%) died after the beginning of the salvage regimen, which precluded a BM assessment to determine response; there were no statistically significant differences between these patients and the rest of the cohort regarding age, indication for treatment, genetic risk, and colonization data. All patients had febrile neutropenia, with admission to the intensive care unit (ICU) in 47% of cases.

By univariate analysis, only age affected the CR rate (*p*=0.045). No significant difference in CR rate was found between the two protocols (MEC 53.5 *vs*. FLAG-IDA 43.7%, *p*=0.447). Furthermore, there were more refractory patients in the FLAG-IDA arm (37.5% *vs*. 4%, *p*=0.02), but more patients died early in the MEC arm (35.7% *vs*. 18.7%, *p*=0.137), even though the latter was not statistically significant. After correcting the initial differences between the two groups regarding indication for salvage through a propensity score calculation (Appendix), a post-matching cohort with 44 subjects was found. In this cohort, no difference in the refractoriness rate could be detected (*p*=0.077).

In the whole cohort, 17 patients proceeded to allogeneic SCT, 15 in CR/CRi and 2 with active disease, with no significant difference in the SCT execution rate between the two groups (*p*=0.470). Only 4/17 transplanted patients were alive by the time of this evaluation. In total, 25% of patients received a second salvage regimen after relapse or refractoriness, and a minority received 3 or 4 different salvage regimens for refractory disease. In the total cohort, 12% of patients died in remission from infection, and IFI was documented in 12% of subjects.

**Figure 1:**
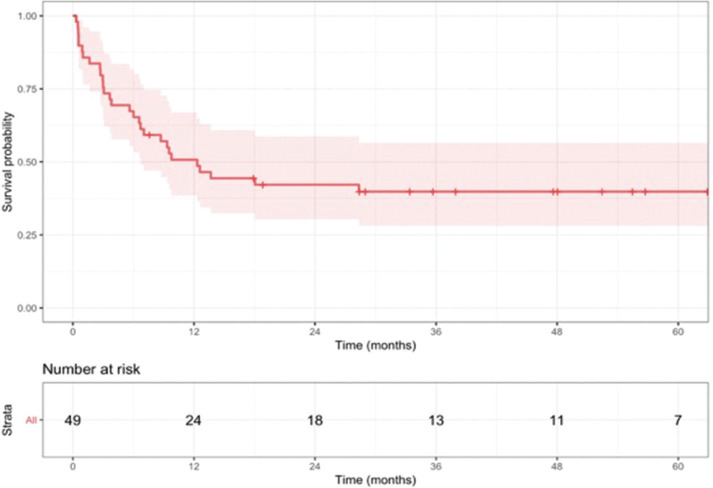
Overall survival curve for the total cohort (n=60).

**Figure 2:**
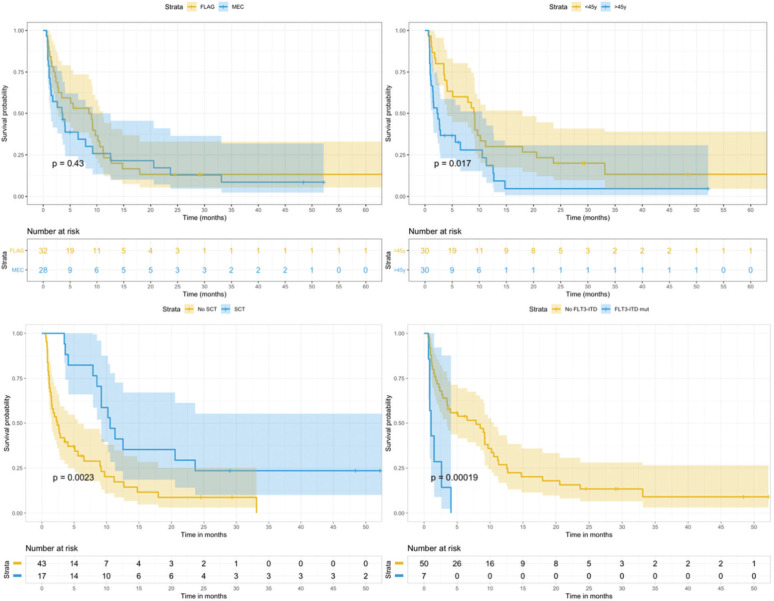
Comparison of overall survival curves according to regimens (top left), age (top right), SCT execution (bottom left), and *FLT3* status (bottom right).

The median follow-up was 48 months (range, 0–85). The median survival in the total cohort was 4 months (95% CI, 2.7–9.2), with a 3-year OS rate of 9.7% (95% CI, 4–23.7) and a 3-year EFS rate of 7.5% (95% CI, 2.5–22.4) (Figure 1). In the univariate analysis, age (*p*=0.02), *FLT3* status (*p*<0.001), and SCT procedure (*p*=0.002) were significantly associated with OS. The chosen regimen did not influence OS or EFS in our analysis and had no influence on the genetic risk, colonization, or time of relapse (Figure 2). The 3-year CIR in patients who responded to the salvage regimen was 30.8% (95% CI, 18–45), with age being the only significantly associated factor (*p*=0.019).

In a multivariable model for EFS that included age, *FLT3* status, and SCT procedure, only the last two indicators remained significant: *FLT3-ITD* mutation (hazard ratio [HR]=4.6 [95% CI, 1.9–11.4], *p*<0.001) and SCT procedure (HR=0.43 [95% CI, 0.22–0.82], *p*=0.01).

## DISCUSSION

In this manuscript, we reviewed the medical charts of 60 patients with r/rAML treated with two standard regimens, MEC and FLAG-IDA, at our center. As expected, a greater proportion of patients with intermediate-high risk disease at diagnosis was included, especially those with a high proportion of *NPM1* plus *FLT3-ITD* mutations. Admittedly, few patients with the *FLT3-ITD* mutation achieve long-term survival even when allogeneic SCT is performed after the first CR, therefore enriching the r/rAML population in this AML subset. This is especially true in centers where FLT3 inhibitors are not yet available 18.

In our study, patients who did not achieve CR at the end of the induction phase or at a least partial response in the early BM assessment were considered refractory and accordingly received a salvage regimen. Currently, in an attempt to standardize further studies addressing this question, recommendations from the European Leukemia Net (ELN) 19 expert panel have defined refractory AML as failure to achieve CR following exposure to at least two courses of intensive induction therapy. In this guide, it was also suggested that the second intensive regimen should ideally include high-dose cytarabine, which is in line with the most recent National Comprehensive Cancer Network (NCCN) guidelines 19,20. Although a significant proportion of patients achieve a CR after a second course of chemotherapy, it is not clear whether those patients have the same prognosis as those who received only one course of chemotherapy 6. Ferguson et al. 21 examined this issue in a retrospective analysis of 8907 patients included in UK Medical Research Council (MRC) trials and found that patients who achieved CR after one course of chemotherapy did considerably better than those who required a second cycle (5-year OS 40 *versus* 8%–21%, *p*<0.0001). Furthermore, a survival difference was also noted between those with PR and truly refractory patients (*p*<0.0001). Achievement of PR was recently reiterated as a significant prognostic factor by Fleming et al. 22 in an Australian cohort. Although it is a debatable definition in AML, it seemed reasonable to include refractory patients in this analysis since they also have a poor prognosis in the majority of cohorts 19,22.

Several regimens of conventional chemotherapy have been studied for r/rAML over the last few decades, encompassing high-dose cytarabine in combination with etoposide, purine analogs, mitoxantrone, or anthracyclines, with variable responses reported 6,23-25. The CR rate reported in our study is similar to that reported for most regimens in r/rAML, especially in retrospective cohorts 23. Several phase II trials testing the feasibility and safety of salvage combinations have been conducted in recent years, many of which did not report any remarkable results (CR ranging from 12% to 89%) 6. It is important to mention that this type of study may be limited by biased selection since a fraction of r/rAML patients are not eligible for intensive chemotherapy according to the formal criteria 23. A noticeable early mortality rate was found in the current study, higher than that reported in previous studies on MEC or FLAG-IDA (4%–12%) 6,15,26-30. A recent study using mitoxantrone in combination with FLAG showed an outstanding CR rate of 73%, raising issues on the choice of anthracycline for r/rAML treatment 29. Greater treatment-related toxicity has already been reported in patients with acute leukemia in Latin America, even though no previous study has addressed this subgroup of adult patients with refractory or relapsed disease in Latin America 5,31-34.

By comparing the CR and survival endpoints between MEC and FLAG-IDA, no remarkable difference was noticed in CR and EFS, with only the *FLT3-ITD* status and execution of SCT remaining as significant factors for survival in our cohort. The former is an important marker of dismal prognosis in AML, even when combined with NPM1 mutation 19. Although *FLT3* mutation was not screened at relapse in our cohort, in most patients with an *FLT3-ITD* mutation at diagnosis, the mutation is retained at relapse, with a higher allelic burden at relapse than at diagnosis 35. This finding reflects the clonal nature of AML and the progressively acquired resistance to conventional chemotherapy in this setting. *FLT3-ITD* mutation was also strongly correlated with survival in other studies on r/rAML, and has been increasingly incorporated into prognostic scores 8,36-38. The current development of FLT3 inhibitors will probably change this scenario since they have already proven their clinical activity in the relapsed or refractory setting, as well as in the first-line setting 18.

Schlenk et al. 38 evaluated prognostic baseline features in 1307 adult relapsed AML patients enrolled on prospective trials of the German-Austrian AML Study Group (AMLSG) between 1993 and 2009. In this study, the duration of the first CR, allogeneic SCT during first-line therapy, age, and *FLT3-ITD* were reported as unfavorable factors for survival, whereas higher duration of the first CR, biallelic CEBPA mutation, core binding factor AML, and allogeneic HCT as treatment of relapse were favorable parameters for survival after relapse 38. Previous studies have also emphasized the role of cytogenetics and CR duration for long-term survival in r/rAML patients 39.

Previous attempts at finding differences among salvage regimens for r/rAML have mostly failed, especially when only conventional chemotherapy is used 6,7,23,24. A recent retrospective publication by AMLSG involving 1025 AML patients with induction failure showed an improved CR rate in patients who received a salvage containing gemtuzumab ozogamicin (odds ratio=0.75, *p*<0.0001) 8. Other innovative approaches for r/rAML include CPX-351, isocitrate dehydrogenase inhibitors, venetoclax, and hypomethylating agents, even though established approaches and combinations are still not accurately defined 23,24.

## CONCLUSIONS

In this analysis, there was no difference in outcome according to the salvage regimen for AML, even though a slightly higher refractoriness rate could be seen in the FLAG-IDA arm. Furthermore, high early toxicity was found, emphasizing the role of supportive care and judicious selection of patients for intensive salvage therapy in this setting. *FLT3-ITD* mutation and SCT remained as significant factors for survival in our study, which is in line with previous studies.

## AUTHOR CONTRIBUTIONS

Silva WF acquired and analyzed the data and wrote the manuscript. Rosa LI helped acquiring data and revising the manuscript. Seguro FS and Silveira DRA helped designing the study and acquiring data. Buccheri V and Bendit I critically revised the manuscript. Velloso EDRP contributed to the study conception and critically revised the manuscript. Rocha V and Rego EM revised the manuscript.

## Figures and Tables

**Table 1:** Baseline characteristics of the total cohort (n=60).

Age (median, range, IQR)	45.5 (17−69, 33.7−54)
Sex	Female (51.7%)
WBC (×10^9^/L) (median, range, IQR)	17.4 (0.58−409.4, 3.3−61.2)
Conventional karyotype	Diploid - 43.3%
	Abnormal - 50%
	No metaphasis - 6.7%
Secondary to MDS or MPN	6.7%
Classification (%)	NOS - 47%
	*NPM1* mut - 22%
	*RUNX1-RUNX1T1* - 5%
	*CBFB-MYH11* - 3%
	*KMT2A* rearrangement - 3%
	5 or 7 abnormalities - 10%
	Complex karyotype (≥3 chromosomal abnormalities) - 7%
	Philadelphia *de novo* - 3%
Genetic risk	Favorable - 18.3%
	Intermediate - 51.6%
	High - 30.1%
*FLT3* status (%) (missing=3.5%)	Wild - 77.2%
	ITD - 12.3% (25)
	TKD - 10.5% (5)
CNS disease (%) (missing=17, 28.3%)	Positive - 3 cases (7%)
Previous induction	Daunorubicin 60 mg/m^2^ - 66.7%
	Daunorubicin 90 mg/m^2^ - 21.7%
	Idarubicin 12 mg/m^2^ - 8.4%
	Mitoxantrone - 3.3%
Indication for salvage treatment	Refractory - 43.3%
	Early relapsed (<1 y) - 45%
	Late relapsed (≥1 y) - 11.7%
Previous SCT	3 pts
HIV infection	2 pts

**Table 2:** Comparison of baseline characteristics between the two groups.

	MEC (n=28)	FLAG-IDA (n=32)	*p*
Age (median)	46	45.5	0.432
Sex	Female (57%)	Female (47%)	0.427
WBC (×10^9^/L) (median)	15.7	28.3	0.366
KPC colonization (%)	50	68.4	0.466
Genetic risk (%)			0.691
*FLT3* status (%) (missing=3, 5%)			0.689
Previous induction			0.775
Indication for salvage treatment	Refractory - 28.5%	Refractory - 56.2%	**0.029**
	Early relapsed (<1 y) - 53.5%	Early relapsed (<1 y) - 37.5%	
	Late relapsed (≥1 y) - 18%	Late relapsed (≥1 y) - 6.3%	
Allo-SCT procedure	8/28 (28.5%)	9/32(28.1%)	0.470

## APPENDIX

**Table:** **Supplementary Table -** Comparison of baseline characteristics between the two groups in the post-matched cohort.

	MEC (n=22)	FLAG-IDA (n=22)	*p*
Age (median)	46.5	43	0.264
WBC (x10^9^/L) (median)	46.4	15.7	0.146
FLT3 status (%)	9	18	0.689
