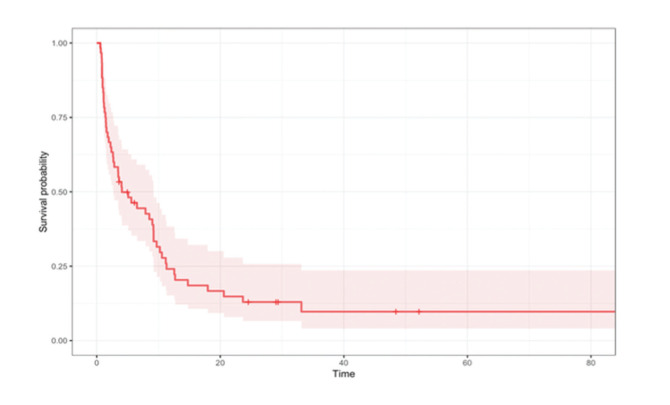# Erratum for: Salvage treatment for refractory or relapsed acute myeloid leukemia: a 10-year single-center experience

**DOI:** 10.6061/clinics/2020/e1566err

**Published:** 2020-08-28

## CLINICS 2020;75:e1566

In the article **Salvage treatment for refractory or relapsed acute myeloid leukemia: a 10-year single-center experience**

Replace **Figure 1** for:

**Figure:**